# Understanding the Interplay between Air Pollution, Biological Variables, and Major Depressive Disorder: Rationale and Study Protocol of the DeprAir Study

**DOI:** 10.3390/ijerph20065196

**Published:** 2023-03-15

**Authors:** Elisa Borroni, Angela Cecilia Pesatori, Guido Nosari, Paola Monti, Alessandro Ceresa, Luca Fedrizzi, Valentina Bollati, Massimiliano Buoli, Michele Carugno

**Affiliations:** 1 EPIGET Lab, Department of Clinical Sciences and Community Health, University of Milan, Via San Barnaba 8, 20122 Milan, Italy; elisa.borroni@unimi.it (E.B.); angela.pesatori@unimi.it (A.C.P.); paola.monti@unimi.it (P.M.); valentina.bollati@unimi.it (V.B.); 2 Occupational Health Unit, Fondazione IRCCS Ca’ Granda Ospedale Maggiore Policlinico, Via san Barnaba 8, 20122 Milan, Italy; luca.fedrizzi@policlinico.mi.it; 3 Department of Pathophysiology and Transplantation, University of Milan, Via Francesco Sforza 35, 20122 Milan, Italy; guido.nosari@unimi.it (G.N.); alessandro.ceresa@unimi.it (A.C.); massimiliano.buoli@unimi.it (M.B.); 4 Department of Neurosciences and Mental Health, Fondazione IRCCS Ca’ Granda Ospedale Maggiore Policlinico, Via Francesco Sforza 35, 20122 Milan, Italy

**Keywords:** major depressive disorder, air pollution, particulate matter, nitrogen dioxide, sulfur dioxide, ozone, DNA methylation, clock genes, inflammatory markers, hormones

## Abstract

Major depressive disorder (MDD) is a serious and disabling condition, whose etiological mechanisms are not fully understood. The aim of the DeprAir study is to verify the hypothesis that air pollution exposure may exacerbate neuroinflammation with consequent alterations in DNA methylation of genes involved in circadian rhythms and hormonal dysregulation, resulting in the worsening of depressive symptoms. The study population consists of 420 depressed patients accessing the psychiatry unit of the Policlinico Hospital (Milan, Italy), from September 2020 to December 2022. Data collection is still ongoing for about 100 subjects. For each participant demographic and lifestyle information, depression history and characteristics, as well as blood samples, were collected. MDD severity was assessed through five rating scales commonly used in clinical practice to assess the severity of affective symptoms. Exposure to particulate and gaseous air pollutants is assigned to each subject using both air pollution monitoring station measurements and estimates derived from a chemical transport model. DeprAir is the first study investigating in a comprehensive picture whether air pollution exposure could be an important modifiable environmental factor associated with MDD severity and which biological mechanisms mediate the negative effect of air pollution on mental health. Its results will represent an opportunity for preventive strategies, thus entailing a tremendous impact on public health.

## 1. Background

Major depressive disorder (MDD) is a serious and disabling condition that, if not promptly managed, is associated with poor quality of life and high social costs [1,2]. Lifetime prevalence of MDD ranges from 2 to 21%, with the highest rates detected in some European countries, also as a result of population ageing [3]. As a matter of fact, people over the age of seventy have a prevalence of this disorder greater than 6% [4]. MDD is twice more common in females than males [5], but completed suicide is more frequent in these latter who have also a minor response to antidepressant treatments [6,7]. In 2019, depression was the second-leading cause of disability worldwide [8].

Despite the high social impact of this condition, the etiological mechanisms underlying MDD are not fully understood. The most accredited etiological model contemplates the contribution of environmental and psychosocial factors in individuals biologically predisposed to develop a mood disorder [9].

MDD is associated with abnormalities of several biological systems resulting in a dysregulation of neurotransmitters, especially of serotonin and noradrenalin [10]. In this regard, the genetic variants predisposing to MDD refer to the serotonin receptors or transporters [11], but also to genes that regulate systems outside the central nervous system (CNS), such as the skeletal one [12], thus explaining the systemic nature of the disorder and the vulnerability of depressed subjects to different medical illnesses [13]. In addition, single nucleotide polymorphisms in clock genes (i.e., those regulating sleep-wake cycles) confer a higher risk of MDD [14].

As MDD and associated medical conditions (e.g., being overweight) share alterations in circadian rhythms [15], recent research has focused on the epigenetic mechanisms underpinning appetite and sleep dysregulation in depressed patients [16]. Circadian rhythms are regulated by the endogenous cellular clock, located in the suprachiasmatic nucleus of the hypothalamus [17]. Moreover, this master clock is regulated at molecular level by complex mechanisms involving positive and negative transcriptional/translational feedback loops, which drive the circadian rhythmicity of “clock gene” transcripts [18]. The positive feedback usually acts during the daytime and the negative one during the night. In the positive one, *CLOCK* and *BMAL1* genes (as a protein dimer complex) are the principal activators of transcription, initiating the molecular circadian cycle with the activation of *PERIOD* (*PER 1-2*) and *CRYPTOCHROME* (*CRY 1-2*) genes. In the negative feedback, when *PER* and *CRY* genes are translated into proteins in the cytoplasm, these enter the nucleus to inhibit their own transcription by binding to the CLOCK–BMAL complex [19]. In addition to these transcriptional mechanisms, it is known that the regulation of these genes is mainly due to DNA methylation, a molecular mechanism of gene expression regulation, which is able to react and be reprogrammed by environmental stimuli [20].

On the other hand, the clock genes regulate the transcription of glucocorticoid receptors [21]. This biological mechanism explains why patients affected by MDD show a paradoxical state of chronic systemic over-inflammation in presence of increased plasma cortisol levels as a result of resistance to the effects of this hormone [22]. Of note, different authors reported that plasma levels of cytokines belonging to innate and adaptive immunity are increased in depressed patients compared to healthy controls and that antidepressant treatment can normalize the levels of these inflammatory factors [23]. Over-inflammation, in turn, triggers oxidative stress [24] and the activation of the hypothalamus–pituitary–adrenal (HPA) axis and the related hypercortisolemia typical of subjects affected by MDD [25].

Different environmental and psychosocial factors were reported to contribute to the onset of MDD, including obstetric complications [26] and childhood trauma [27]. In the last decade, air pollution has been hypothesized as a potential contributor of the onset of MDD, also in the light of the increasing mental health social costs due to urbanization [28]. Convincing data show that several air pollutants may be implicated in the onset or the worsening of depressive symptoms [29]. Of note, air pollution may exacerbate depressive symptoms by different biological mechanisms: (i) increasing systemic inflammation that in turn modifies neurotransmitter release and alters circadian rhythms [30], (ii) overcoming the blood–brain barrier and having a direct toxic effect on the CNS [31], and (iii) stimulating brain microglia by changes in bone marrow of the skull activated by chronic peripheral damage (e.g., in the respiratory system) [32].

## 2. Aims and Hypotheses

**Figure 1:**
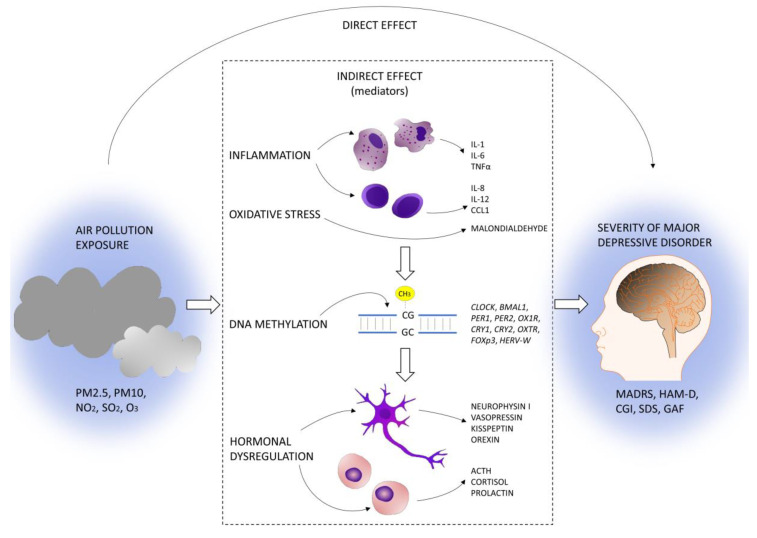
DeprAir conceptual framework.

Given the above summarized available evidence, the relationships between air pollution exposure and inflammation, clock gene methylation, and hormonal dysregulation appear a promising mechanism for explaining MDD development and worsening. Our hypothesis is that air pollution exposure may exacerbate neuroinflammation with consequent epigenetic and hormonal dysregulation, resulting in the worsening of depressive symptoms (Figure 1).

To verify our hypothesis, we will follow a multi-step approach within a cross-sectional study aimed to

1. investigate the association between short-term exposure to air pollution and MDD severity in a sample of patients with MDD;
2. assess the relationship between MDD severity and different biological (inflammatory, epigenetic, hormonal) markers, measured in blood samples collected from all the subjects recruited in step 1;
3. evaluate the association between air pollution and the biological variables of interest identified in step 2;
4. quantify the specific contribution of the investigated biological variables in the chain of events linking air pollution exposure to MDD severity through a mediation analysis.

We hereby present study design, field activities, management organization, and characteristics of study subjects for which data collection has been completed.

## 3. Materials and Methods

### 3.1. Study Design

The DeprAir study is a cross-sectional study conducted in the Lombardy region, Italy, whose aim is to understand the interplay between air pollution, biological variables, and MDD.

Lombardy is located in the northern part of Italy, covering an area of 23.864 km^2^ and with a resident population of about 10 million people (https://bit.ly/3kXL6NZ, accessed on 13 February 2023). It is composed of 12 provinces, among which Milan is the capital, with around 1.3 million residents (https://bit.ly/3HsVogO, accessed on 13 February 2023).

### 3.2. Study Population

The study population consists of 420 depressed patients accessing the psychiatry unit of the Policlinico Hospital in Milan (Italy), from September 2020 to December 2022. Data collection is still ongoing for about 100 subjects, while it has been completed for 317 subjects (75% of the target population), recruited up to 1 July 2022. Participants are recruited by trained psychiatrists among hospitalized or day-hospital patients or outpatients, who have been accessing the hospital since 2003 for MDD. The physician contacts already known patients by phone or meets them in person, in case they are hospitalized or outpatients, describes the study aims, and asks for participation in the study. To be eligible, patients must fulfill the following criteria: being ≥18 years old at enrollment; having received a diagnosis of MDD and having signed the consent form. Patients are excluded when they: have a medical condition associated to behavioral disorders (e.g., unbalanced hypothyroidism or stroke); have abused of drugs in the last four weeks; have comorbidities related to other psychiatric disorders (except for personality disorders different from borderline personality disorder); have medical conditions which may alter inflammatory markers (e.g., autoimmune diseases); have known ongoing infections; are taking treatments which may influence biological markers of interest (e.g., corticosteroids or interferons); are pregnant; are <18 years old. The participation rate in the study period was 75%.

### 3.3. Epidemiological and Clinical Data Collection

At recruitment, each enrolled subject is asked to sign a consent form to:

- extract personal information from medical records (if already known);
- answer two questionnaires administered by the psychiatrist to collect demographic and lifestyle information, as well as depression history and characteristics;
- donate 30 mL of blood (five EDTA tubes of 6 mL each).

### 3.4. Questionnaire on Sociodemographic and Lifestyle Characteristics

Each patient is interviewed by the psychiatrist who fills in the questionnaire. The questionnaire includes information on sociodemographic data (date of birth, sex, height, weight, education, occupation status), recent residential history (current complete address, previous complete address if changed in the last year, traffic status in the residential area), smoking history, including passive smoking at home and at workplace (smoking status; duration of smoking; number of cigarettes smoked; age at starting; age at quitting if former smoker; number of smoking family members; number of smoking colleagues at work), current health status including information on history of selected diseases (hypertension, hypercholesterolemia, diabetes, cancer, heart disease, renal failure) and medication, physical activity levels and sedentary behavior, type of diet (eating everything, vegetarian, vegan), and drinking habits (how much tea, coffee, wine, beer, and spirits).

Table 1 summarizes the main demographic and lifestyle characteristics of the study population. MDD patients, recruited up to 1 July 2022, have a mean age of 51.5 years and are primarily composed of females (67.2%). The largest majority has a high school or a university degree (78.6%), is employed (42.9%), and has been recruited as either new (38.2%) or already known outpatients (27.8%). About 29% of the entire population is currently smoking, while 13% is represented by former smokers.

### 3.5. Questionnaire on History and Characteristics of Depression

The anamnestic questionnaire collects information about depression history and characteristics, in particular: family psychiatric history [including the type(s) of psychiatric disorder(s)], age at onset, duration of untreated illness in months, total duration of illness in years, duration of the latest episode in months, number of depressive episodes, hospitalizations (no vs. yes + total number of hospitalizations), suicide attempts (no vs. yes + total number of suicide attempts), psychotic symptoms (no vs. yes), seasonality of depression (no vs. yes), subtype of depression (melancholic, psychotic, with strong symptoms of anxiety, atypical), history of lifetime substances abuse (never, single-abuse or multiple-abuse and, if the subject ever suffered of substances abuse, type(s) of abuse(s) from alcohol, cocaine, cannabis, heroin, LSD, amphetamines, drugs, and MDMA), antidepressant treatment (no vs. yes + type of antidepressant assumed, active principle, dose, number of active principles ever assumed, suspension, and other treatments).

Table 2 summarizes MDD characteristics. Mean age at onset of MDD is 39.5 years, mean number of MDD episodes is 2.9, and mean total duration of illness is 11.3 years. About 48.9% of participants has a family history of psychiatric disorders, while 36% has a family history of MDD. Overall, 23% has been hospitalized for MDD, and 15.1% has committed at least one suicide attempt. The largest majority has a MDD with strong symptoms of anxiety (37.2%) followed by the melancholic subtype (36.9%). About one fifth of patients (19%) has suffered from single or multiple substance(s) abuse with alcohol and cannabis being the most frequent addictions. Almost all (88.3%) patients take an antidepressant treatment, with selective serotonin reuptake inhibitors (62.9%) and serotonin and norepinephrine reuptake inhibitors (16.4%) being the most frequent ones.

### 3.6. Diagnostic Criteria and Rating Scales

Diagnoses of MDD are confirmed by using Structural Clinical Interview for DSM-5 (SCID—Italian version) [33]. The psychiatrist evaluates depression severity of recruited patients by administering them the following rating scales, which are commonly used in clinical practice to assess the severity of affective symptoms:

- Montgomery-Asberg Depression Rating Scale (MADRS): it is a tool used to assess core symptoms of MDD (e.g., anhedonia). It is composed of the following 10 items: apparent sadness, reported sadness, inner tension, reduced sleep, reduced appetite, concentration difficulties, lassitude, inability to feel, pessimistic thoughts, suicidal thoughts. Each item has a severity scale from 0 to 6, with higher scores reflecting more severe symptoms. Ratings can be summarized in an overall score (from 0 to 60), which allows to stratify severity of depression as: 0–6: no depression, 7–19: mild depression, 20–34: moderate depression, ≥35: severe depression [34];
- Hamilton Depression Rating Scale (HAM-D) 21-item: this tool is indicated to assess anxiety and somatization symptoms of MDD. It is composed of 21 questions on types of symptoms associated with depression such as anxiety, mood, insomnia, and somatic symptoms experienced within the past week. Each symptom is rated on a scale of 0–2, 0–3, or 0–4 with 0 being absent and 2, 3, or 4 being the most severe. To obtain the overall score of severity (from 0 to 67), ratings can be added, and the total score can be stratified as: 0–7: no depression, 8–16: mild depression, 17–23: moderate depression, ≥24: severe depression [35];
- Clinical Global Impression-severity of illness (CGI): this tool is used by the psychiatrist to evaluate the global severity of illness answering the following question: “Considering your total clinical experience with this particular population, how mentally ill is the patient at this moment?”. The answer is given following this seven-point rating scale: 1 = normal, not at all ill; 2 = borderline mentally ill; 3 = mildly ill; 4 = moderately ill; 5 = markedly ill; 6 = severely ill; 7 = among the most extremely ill patients [36];
- Sheehan Disability Scale (SDS): this scale is used to evaluate the social dysfunction associated with MDD. It consists of a self-reported assessment of functional impairment composed of five items. The first three are global rating scales which assess impairment in work, home, and family responsibilities. There are two additional questions which measure perceived stress and social support. The items are scored individually on 10-point numerical rating scales, except for the “social support” one that can be scored 0–100 [37];
- Global Assessment of Functioning (GAF): this tool is used to evaluate the overall impairment associated with MDD. In particular, it measures how much a person’s symptoms affect his/her day-to-day life on a scale of 0 to 100. This scale is broken into 10 sections, which are known as anchor points. The higher the score is, the better the patient is able to handle daily activities, suggesting that a lower score indicates a greater social disfunction associated with depression [38].

Summary statistics for severity of MDD scales are reported in Table 3. Scales mean scores are 27.1 for MADRS, 22.7 for HAM-D, and 59.6 for GAF. Based on CGI scores, most of the patients are mildly (22.4%) or moderately (30%) ill, while mean scores for SDS single domains are: 7.2 for work, 6.7 for relationships, 6.6 for family, 6.3 for stress, and 59.8 for social support.

### 3.7. Blood Sample Collection

Specific laboratory standard operating procedures have been developed to ensure quality control of every step involved in biospecimen collection and storage. Blood drawing is performed directly by the psychiatrist. Each subject provides a 30 mL blood sample in five EDTA tubes, which are delivered to laboratory and processed within 4 h. One of the tubes is used for blood cell count, while the remaining ones are centrifuged and processed to obtain plasma and buffy coat fractions. Plasma and buffy coat samples are stored at −80 °C for subsequent quantification of inflammatory and hormonal markers and DNA methylation analysis, respectively.

### 3.8. Inflammatory Markers

As mentioned above, markers of both innate and adaptive immunity have been widely associated with the severity of MDD. Considering the innate immunity, the following markers will be measured—IL-1, IL-6, and TNFα, while the following markers of adaptive immunity will be considered—IL-8, IL-12, and CCL1. In addition, the levels of malondialdehyde will be measured as a parameter of oxidative stress. All these markers will be evaluated on plasma by using ELISA (enzyme linked immunosorbent assay) kits.

### 3.9. DNA Methylation of Clock and Clock-Controlled Genes

We have selected 10 target genes (*CLOCK*, *BMAL1*, *PER1*, *PER2*, *OX1R*, *CRY1*, *CRY2*, *OXTR*, *FOXp3*, *HERV-W*), which include clock genes and genes directly stimulated by clock pathways, to measure DNA methylation by pyrosequencing. Following genomic DNA extraction from buffy coat, we performed this using a Promega kit (Madison, WI, USA), 3 μg DNA (concentration 25 ng/μL), which will be bisulfite-treated using EZ DNA Methylation-Gold™ Kit (Zymo Research, Orange, CA, USA) according to the manufacturer’s protocol. Bisulfite-treated DNA will be stored at −20 °C and used shortly after treatment. For each reaction, a 50 μL PCR will be carried out by adding 10 μL of bisulfite-treated genomic DNA to 25 μL of GoTaq Green Master mix (Promega, Madison, WI, USA), 1 μL of forward primer (10 μM), 1 μL of reverse primer (10 μM), and water. One of the primers is biotin-labelled and is used to purify the final PCR product by Sepharose beads. The PCR product will be bound to Streptavidin Sepharose HP (Amersham Biosciences, Uppsala, Sweden), and the Sepharose beads containing the immobilized PCR product will be purified, washed, denatured using a 0.2 M NaOH solution, and washed again using the Pyrosequencing Vacuum Prep Tool (Pyrosequencing, Inc., Westborough, MA, USA), as recommended by the manufacturer. Then, 0.3 μΜ Pyrosequencing primer will be annealed to the purified single-stranded PCR product, and Pyrosequencing will be performed using the PyroMark Q96 MD Pyrosequencing System (QIAGEN). Methylation quantification will be performed using the provided software (Pyro Q-CpG software, version 1.0.9—Biotage, Uppsala, Sweden)). The degree of methylation will be expressed as percentage of 5-methylated cytosines (%5mC) over the sum of methylated and unmethylated cytosines. We will use built-in controls to verify bisulfite conversion efficiency.

### 3.10. Hormonal Markers

Hormonal changes, as well as inflammation, can be correlated with the severity of MDD. The following hormones (including neuropeptides) will be measured: adrenal corticotropic hormone (ACTH), cortisol, neurophysin I (a good marker of oxytocin levels in the CNS), vasopressin, kisspeptin, orexin, and prolactin. Plasma samples of the recruited subjects are collected at similar time (around 11 a.m., as hormone levels change according to circadian rhythms) and measured using ELISA kits.

### 3.11. Exposure Assessment

**Figure 2:**
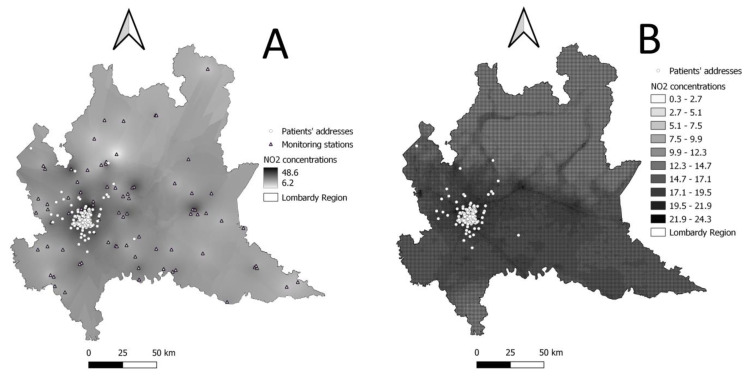
Graphical representation of air pollution concentration levels (NO_2_ annual average for year 2021 chosen as an example). (**A**) Point measurements from monitoring stations expanded to the whole Lombardy territory through ordinary Kriging (used for graphical purposes only). (**B**) NO_2_ concentrations predicted by FARM model.

As air pollutants of interest, we consider particulate matter with diameter less than or equal to 10 (PM10) and 2.5 µm (PM2.5), nitrogen dioxide (NO_2_), sulfur dioxide (SO_2_), and ozone (O_3_). Each patient’s residential address is translated into spatial coordinates using the web tool GPS Visualizer (https://www.gpsvisualizer.com/, accessed on 17 January 2023) and geocoded using QGIS (QGIS Development Team, 2022. QGIS Geographic Information System. Open-Source Geospatial Foundation Project. http://qgis.osgeo.org, accessed on 17 January 2023). Air pollution levels are assigned to each patient following two approaches (Figure 2):

(i) PM10, PM2.5, NO_2_, SO_2_, and O_3_ measurements retrieved from the air quality monitoring stations of the Regional Environmental Protection Agency (ARPA Lombardia). Daily means of pollutant levels measured at the station closest to the subject’s residential address are assigned to each subject. Missing values for each pollutant on a specific day and monitor are imputed by computing the average of measurements of that pollutant for the previous and the following seven days.
(ii) PM10, PM2.5, NO_2_, and O_3_ daily mean estimates are derived from the Flexible Air quality Regional (FARM) model [39,40]. This type of Eulerian model takes into account the atmospheric chemistry, together with transport, dispersion, and deposition phenomena [41,42]. By integrating data measured from ARPA air quality and meteorological monitoring stations, emissions, concentrations at the beginning of the simulation period, and trend in adjacent areas, it estimates pollutants’ concentrations as daily/hourly means covering the whole Lombardy territory with a grid of 1 × 1 km cell. Each subject is assigned the daily average of pollutants’ exposure estimated inside the grid cell where his/her residential address fell.

Given the finer spatial resolution of its estimates, we will give priority to the second approach. We will make use of data from monitoring stations, should the FARM model estimates not be available.

Meteorological data (e.g., temperature, humidity) are retrieved from ARPA monitoring stations too.

To take into account the potential effect of short- and medium-term exposures, we will investigate several time windows: (i) single daily lags obtained considering pollutants’ daily means from the day of recruitment (lag0) up to 30 days before (lag30), (ii) averaged daily lags obtained by averaging pollutants’ levels of the day of recruitment with the levels of the day before (lag01) and of each preceding day up to 30 days before (lag030). Exploratory analyses will also be performed to investigate time windows potentially representative of cumulative exposures (e.g., annual averages).

### 3.12. Ethical Issues

The study design, research aims, and measurements have been approved by the local Institutional Review Board of the Fondazione IRCCS Ca’ Granda Ospedale Maggiore Policlinico (approval numbers 498_2020bis and 950_2020). Participants agreed to sign a written informed consent explaining the study in detail, as well a consent for biobanking of the blood samples for future research studies. In any case, new measurements will only be performed after approval of the Ethical Committee.

### 3.13. Statistical Analysis and Power Calculation

We will use standard descriptive statistics to summarize data. Graphical inspection of the main variables of interest will be performed to examine their distribution and uncover the need for transformation.

Associations between concentration levels of air pollutants and MDD severity, and between air pollution and biological variables, will be assessed by multiple linear regression models, adjusted for main confounders of interest (e.g., age, sex, socioeconomic status, smoking habit, season, temperature, etc.). Potential non-linearity of the associations will be verified flexibly modelling air pollution variables as splines with various knots depending on their distribution. Results will be expressed either as regression coefficients (β, which will take the unit of measure of the outcome variable, e.g., rating scale scores) or as percent change in the investigated outcome, with corresponding 95% Confidence Intervals (95%CI) for a given variation in air pollutant exposure levels.

We will perform similar models with biological variables used as independent variables to assess the association between biological variables and MDD severity. This will allow us to evaluate a pool of biological markers, which might predict MDD severity.

Sensitivity analyses will be also performed, e.g., considering duration of illness (both treated and untreated) and age at onset or stratifying by depression subtype to verify its potential for effect modification.

To investigate potential pathways that could explain the observed associations between exposure to air pollutants and MDD severity, we will perform a mediation analysis, an approach which allows to examine how intermediate variables (i.e., the mediators) are related to the observed exposure-outcome relationship [43].

As our study will test several associations, a formal power calculation was not possible, and sample size was calculated on the basis of the primary hypothesis that short-term exposure to air pollution is associated with MDD severity. We based our sample size calculation on preliminary results on 195 women randomly selected from the SPHERE population [44], for which we observed a positive association between the Beck Depression Inventory (BDI) score and the average exposure level of PM10 in the third day preceding the day of recruitment. A sample size of 420 achieves 98% power to detect a change in slope from 0.09 (under the null hypothesis) to 0.19 (under the alternative hypothesis) when the standard deviation of the PM10 variable distribution is 17 μg/m^3^, the standard deviation of the BDI variable distribution is 9, and the two-sided significance level is 0.05. These data represent an informative source for our study, as the BDI scale can be converted into the HAM-D scale (which we administer) and vice versa, with equipercentile linking [45]. Based on our previous experience on studies on health effects of air pollution exposure (e.g., [46,47]) and clinical practice on depressed patients, as well as on some of the characteristics of the participants enrolled so far, we expect that the distribution of the variables in our study participants will resemble those of our preliminary data.

### 3.14. Data Management and Privacy Protection

To protect each patient’s privacy, all collected information and biological samples are anonymized from personal identifying information and each participant can be identified only through a five-digit randomly assigned barcode. The information linking each subject’s identity to his/her personal barcode is held in a secure database. Questionnaire data are collected in paper forms and are subsequently imputed in the database, regularly checking quality and completeness of information.

Data processing is anonymous, and the highest level of confidentiality is assured for all personal information. Quality of collected data is routinely checked by comparing information from different sources (clinical records, questionnaires, biochemical exams), assessing variable range and distribution, and verifying database completeness through simple statistics.

### 3.15. Dissemination of Results

A study website has been created (https://deprair.com/, accessed on 10 February 2023) and will be updated regularly. It contains relevant information about the study and related events. Results and dissemination material will be published on the website, at the time they will be produced. Furthermore, results will be converted into user-friendly materials and published in press releases, educational programs, as well as scientific conferences and journals.

### 3.16. Collaboration Opportunities

The rich set of clinical information and molecular data of the DeprAir study makes it a good environment for collaboration opportunities. Proposals from outside the study team for research projects to test specific hypotheses on the DeprAir population will be reviewed by our research group and can be sent to info@deprair.com.

## 4. Discussion

To the best of our knowledge, DeprAir is the first study investigating whether exposure to air pollution could be an important modifiable environmental factor associated with MDD severity and which biological mechanisms mediate the negative effect of air pollution on mental health. We had previously reported that particulate air pollution is associated with a higher risk of manic episodes with mixed features in hospitalized bipolar patients and showed that increased levels of PM move the manic episode towards the depressive pole of the bipolar disorder spectrum [46]. Our current study will thus allow us to further deepen our knowledge in the complex pathway that links environmental exposures to psychiatric disorders.

In particular, DeprAir will clarify the role of exposure to environmental air pollution on MDD severity. This aim will be achieved by considering two methods for environmental exposure, thus maximizing the probability of assigning accurate exposure values to our population. Both approaches have proved their soundness in similar settings, as documented in previous studies on health effects of air pollution exposure [44,46,47,48]. In particular, the assignment of personal exposures based on residential address using daily pollutant levels at a 1 × 1 km resolution represents the best compromise between spatial and temporal resolution that we can currently achieve when considering our entire regional territory. Finally, the association of interest will be studied considering single-exposure models, as well as multiple-exposure techniques, to estimate the most accurate impact of air pollution on MDD. For the same purpose, roles of temperature and humidity in the association between air pollution exposure and MDD will also be evaluated.

The present study will also shed light on the biological mechanisms underlying MDD worsening, assessing whether exposure to air pollution determines alterations in inflammatory, epigenetic, and hormonal variables, as well as identifying a pool of biological markers that might predict MDD severity. In addition, DeprAir will allow to disentangle the role that each element of the hypothesized causal pathway (environmental stressors and inflammatory, epigenetic, and hormonal variables) might play in determining MDD severity through mediation models. To apply these models, some basic conditions have to be met: the association between air pollution exposure and the outcome (MDD severity) is statistically significant; the hypothesized mediators have an effect on the outcome when the exposure is controlled for; air pollution has an effect on the mediators. If these criteria will be satisfied, the mediation analysis will allow to evaluate direct (DE) and indirect effects (IE). DE is the effect of air pollution on MDD severity adjusted for the mediators, while IE estimates the proportion of the effect of air pollution exposure on MDD severity that is mediated by the biological variables. Correlations among variables will be considered through the use of high dimensional mediation analysis.

We are aware that one possible limitation of our study is its limited sample size, which, however, is an intrinsic constraint of studies examining preliminary hypothesis with collection of biological data. Should our hypotheses be confirmed, our findings will have to be validated in larger study populations.

The results of DeprAir will represent an opportunity for preventive strategies, having the possibility to entail a tremendous impact on public health. In particular, the present study represents an opportunity to open new strategies in terms of prevention for MDD: if air pollution will be confirmed as an important environmental risk factor for the severity of depressive symptoms, reduction in the levels of air pollutants may represent an easy strategy to reduce the severity of depressive symptoms with a clear economic saving due for example to reduced psychiatric hospitalizations. Of note, MDD is not only associated with direct costs due to hospitalizations, but also to indirect costs, such as loss of days of work for patients and their caregivers. Caregivers suffer from the high social burden due to MDD, as they often look after their relatives, also in the light of the high risk of suicide associated with the depressive illness. As such, even a small improvement in the management of patients affected by depression could lead to a great advantage in terms of the patients’ quality of life, burden for caregivers, and social costs. In addition, the investigation of the biological factors that potentially mediate the toxic effects of air pollution on the brain may open to new pharmacological strategies that may be represented by anti-inflammatory drugs and regularization of circadian rhythms or of hormone-associated biological cascades. Furthermore, the topic of air pollution and its consequences on mental health can give a further drive towards the realization of towns that include green areas or other facilities aimed to air quality improvement.

## Figures and Tables

**Table 1:** Demographic and lifestyle characteristics of 317 study subjects recruited until 1 July 2022.

Demographic and Lifestyle Characteristics	Mean (SD)/N (%)
Age	51.5 (17.4)
Sex	
Females	213 (67.2%)
Males	104 (32.8%)
BMI	
Underweight	20 (6.3%)
Normal weight	171 (53.9%)
Overweight	83 (26.2%)
Obese	43 (13.6%)
Education	
Primary school or less	14 (4.4%)
Secondary school	54 (17.0%)
High school	152 (48.0%)
University	97 (30.6%)
Occupation status	
Employee	136 (42.9%)
Unemployed	70 (22.1%)
Retired	75 (23.7%)
Other	36 (11.4%)
Smoking status	
Never smoker	185 (58.4%)
Former smoker	41 (12.9%)
Current smoker	91 (28.7%)
Passive smoking exposure	
Yes	95 (30.0%)
No	222 (70.0%)
Residence traffic exposure	
Mild	80 (25.2%)
Moderate	112 (35.3%)
Heavy	125 (39.4%)
Source of recruitment	
Outpatients	121 (38.2%)
Day-hospital	64 (20.2%)
Hospitalizations	44 (13.9%)
Already known outpatients recontacted for the study	88 (27.8%)

**Table 2:** MDD characteristics in 317 study subjects recruited until 1 July 2022.

Major Depressive Disorder Characteristics	Mean (SD)/N (%)
Age at onset of MDD	39.5 (17.6)
Number of MDD episodes	2.9 (2.7)
Family history of psychiatric disorders	
Yes	155 (48.9%)
No	162 (51.1%)
Family history of MDD	
Yes	114 (36.0%)
No	203 (64.0%)
Total duration of untreated MDD in months	22.2 (53.3)
Total duration of MDD in years	11.3 (13.0)
Duration of last MDD episode in months	9.7 (12.6)
Hospitalizations for MDD (N)	
Yes	73 (23.0%)
No	244 (77.0%)
Among those hospitalized for MDD (73), N of hospitalizations	1.5 (0.9)
Psychotic symptoms	
Yes	22 (6.9%)
No	295 (93.1%)
Suicide attempts	
Yes	48 (15.1%)
No	269 (84.9%)
Seasonality of MDD	
Yes	97 (30.6%)
No	220 (69.4%)
Depression subtype	
Melancholic	117 (36.9%)
Atypical	48 (15.1%)
Psychotic	12 (3.8%)
With strong symptoms of anxiety	118 (37.2%)
No prevalent type	22 (6.9%)
Lifetime substances abuse	
Single abuse	47 (14.8%)
Multiple abuse	14 (4.4%)
No	256 (80.8%)
Among ever abusers (61), type(s) of abuse	
Alcohol	28 (45.9%)
Cannabis	25 (41.0%)
Heroine	5 (8.2%)
Cocaine	8 (13.1%)
LSD	1 (1.6%)
Amphetamine	1 (1.6%)
MDMA	0 (0.0%)
Drugs	16 (26.2%)
Any antidepressant treatment	
Yes	280 (88.3%)
No	37 (11.7%)
Among subjects taking treatment (280), type of treatment	
Selective Serotonin Reuptake Inhibitors (SSRI)	176 (62.9%)
Serotonin and Norepinephrine Reuptake Inhibitors (SNRI)	46 (16.4%)
Tricyclics	26 (9.3%)
Bupropion	1 (0.4%)
Mirtazapine	6 (2.1%)
Vortioxetine	7 (2.5%)
Trazodone	11 (3.9%)
Others	7 (2.5%)

**Table 3:** Scales of MDD severity in 317 study subjects recruited until 1 July 2022.

Severity of MDD Scales	Mean (SD)/N (%)
Montgomery-Asberg Depression Rating Scale (MADRS)	27.1 (12.3)
Hamilton Depression Rating Scale (HAM-D)	22.7 (11.3)
Global Assessment of Functioning (GAF)	59.6 (15.1)
Clinical Global Impression (CGI)	
Normal, not at all ill	16 (5.1%)
Borderline mentally ill	36 (11.4%)
Mildly ill	71 (22.4%)
Moderately ill	95 (30.0%)
Markedly ill	53 (16.7%)
Severely ill	38 (12.0%)
Among the most extremely ill patients	8 (2.5%)
Sheehan Disability Scale (SDS)	
Work	7.2 (2.7)
Relationships	6.7 (2.6)
Family	6.6 (2.7)
Stress	6.3 (2.7)
Social support	59.8 (26.9)

## Data Availability

Data can be accessed upon reasonable request to the corresponding author (michele.carugno@unimi.it).
